# Resistance of Synthetic and Biologic Surgical Meshes to Methicillin-Resistant* Staphylococcus aureus* Biofilm: An* In Vitro* Investigation

**DOI:** 10.1155/2019/1063643

**Published:** 2019-03-13

**Authors:** Ethan Kallick, Laura Nistico, Mark Longwell, Barbara Byers, Frank Cartieri, Rachael Kreft, Howard Edington

**Affiliations:** ^1^Department of Surgery, Allegheny Health Network, Pittsburgh, PA, USA; ^2^Center for Excellence in Biofilm Research, Allegheny Health Network Research Institute, Pittsburgh, PA, USA

## Abstract

Surgical meshes have become the standard procedure for a variety of surgical applications with 20 million meshes being implanted each year. The popularity of mesh usage among surgeons is backed by the multiple studies that support its functionality as a tool for improving surgical outcomes. However, their use has also been associated with infectious surgical complications and many surgeons have turned to biologic meshes. While there have been several studies investigating synthetic meshes, there is limited data comparing synthetic and biologic meshes* in vitro *in an infection model. This study evaluates the* in vitro* susceptibility of both synthetic and biologic meshes to single-species methicillin-resistant* Staphylococcus aureus* (MRSA) biofilms. This research compares biofilm biomass, average thickness, and coverage between the three meshes through* florescent in situ hybridization* (FISH), confocal scanning microscopy (CSLM), and image analysis. We also report the varying levels of planktonic and attached bacteria through sonication and cfu counts. While the data illustrates increased biofilm formation on biologic mesh* in vitro*, the study must further be investigated* in vivo *to confirm the study observations.

## 1. Introduction

 Surgical meshes are a relatively recent scientific advancement from the latter half of the 20th century. Meshes have become the standard procedure for a variety of surgical applications, including hernia repair, colorectal surgery, soft tissue reconstruction, and tissue/organ support. With 20 million meshes being implanted worldwide each year [1], meshes have undoubtedly become significant in surgical practice. Since the advent of the first synthetic mesh in 1962 [2], advancements in mesh science have led to the development of a large number of commercially available synthetic meshes with variable characteristics, such as chemical composition, structural design, porosity, filament number, and absorption. More recently, biologic meshes have gained popularity as an alternative to synthetic. Biologic meshes are derived from human, porcine, or bovine tissue and undergo a proprietary process that includes decellularization and sterilization to leave behind a collagen matrix. The acellular, biologic matrix is designed to support tissue remodeling and new collagen deposition as a means to increase tissue ingrowth and fully integrate the mesh within the host tissue.

Indeed, the popularity of mesh usage among surgeons is backed by multiple studies that support its functionality as a tool for improving surgical outcomes, especially with regard to hernia repair. A review of several studies shows that the use of a mesh for the repair of incisional hernias decreased recurrence rates by an average of 30% compared to nonmesh repair [3–5]. Similarly, a multi-trial review conducted by Grant et al. reported that mesh usage has reduced recurrence rates of abdominal hernias to less than 1.5% [6].

While surgical meshes have proven beneficial for a variety of surgical applications, their use has also been associated with significant clinical complications including seroma formation, fistulas, erosion into adjacent structures, chronic pain, and infection [7]. This research focuses on mesh-related infection, which is the most significant clinical complication relating to mesh implantation. Infection has been shown to occur in 1-2% of all cases involving mesh [8, 9] and up to 18% of open incisional hernia repair cases [10].

During the infection of a prosthetic biomaterial, the bacteria become phenotypically different as they begin to colonize the mesh, physically joining together to form a biofilm. As the biofilm matures, it continuously produces an extracellular matrix containing various different extracellular polymeric substances (EPS) such as proteins, DNA, and polysaccharides, which enhances adhesion and forms a structural barrier to the external environment surrounding the biofilm [11, 12]. Meshes containing biofilms are resistant to both the antibiotic therapy and the host immune response, which can necessitate removal of the infected mesh. By interfering with tissue integration and repair, infection has the potential to increase other significant comorbidities such as recurrence, inflammation, adhesion, and even structural loss of the abdominal wall [2].

In mesh infections, the most common recovered microorganism is methicillin-resistant* Staphylococcus aureus* (MRSA) [13, 14]. In one study of mesh-related infections following incisional herniorrhaphy, MRSA infections accounted for 63% of the postsurgical mesh infections [15]. Given its clinical significance, we used MRSA in our investigation of infection susceptibility to single-species biofilm formation for both synthetic and biologic surgical meshes.

While there have been several studies that investigated bacterial attachment and biofilm formation among the various synthetic meshes [7, 16], there is limited data directly comparing synthetic meshes to their biologic counterparts. Furthermore, the tendency for biologic mesh to become quickly integrated into host tissue following implantation is thought to minimize foreign body reaction, improve wound healing, and make the mesh less prone to bacterial adhesion and biofilm formation [17–19]. Hence, we evaluated the capacity of three commercially available meshes to resist MRSA biofilm formation* in vitro*. These meshes, 2 synthetic and 1 biologic, are currently used at our institute for tension-free hernia and other soft tissue repairs.

## 2. Materials and Methods

### 2.1. Bacterial Strain

Methicillin-Resistant* Staphylococcus aureus* CGS.Sa03 [17] was obtained from the Center of Excellence in Biofilm Research at Allegheny General Hospital. The strain was recovered by standard microbiological culture of explanted polypropylene mesh from a patient diagnosed with cellulitis and abscess following mesh placement. The genome of CGS.Sa03 has been previously sequenced and reported [20].

### 2.2. Preparation of Mesh

Three mesh types were used for these experiments: a biologic mesh derived from human dermal collagen (Bard® Davol Inc., Cranston, RI), an absorbable synthetic polyglactin 910 woven mesh (Ethicon Inc., Sommerville, NJ), and a permanent synthetic polypropylene mesh (Bard® Davol Inc., Cranston, RI).

Samples of each mesh type were prepared by aseptically cutting squares (1 × 1 cm) from the material. The mesh squares were then placed in 35 mm polystyrene Petri plates. Twelve samples of each mesh type were prepared. This experiment was performed in triplicate and was completed three times per mesh.

### 2.3. Mesh Inoculation

The strain of MRSA was recovered from −80°C storage and streaked to isolation on Brain Heart Infusion (BHI) plates with overnight incubation (37°C, 5% CO_2_). An individual colony was then placed into 10 mL of BHI broth (Gibco, Life Technology Corp., Grand Island, NY) and incubated in a 37°C shaker at 50 rpm for 2.5 hours. The inoculated broth culture was then diluted in BHI to achieve an inoculum dose of 10^2^ CFU mL^−1^, which was then applied to half of the samples (n=6). The remaining samples (n=6) served as controls and received 4 mL of sterile BHI. All twelve plates were then incubated in a 37°C shaker at 50 rpm for 24 hours, at which point biofilms were examined. Biofilm growth was analyzed by confocal microscopy using LIVE/DEAD® Baclight™ Bacterial Viability stain (Invitrogen Detection Technologies, Eugene, OR) and quantified by Comstat Image Analysis and the enumeration of colony-forming units (CFUs) after biofilm detachment.

### 2.4. Biofilm Analysis by Confocal Microscopy

After 24 hours of incubation, six plates containing bacterial inoculums samples (n=3) and control samples (n=3) were removed for confocal imaging. Confocal images were taken in triplicate. The media in the plate were removed by pipette and each sample was rinsed twice with 5 mL of sterile Hank's balanced salt solution (HBSS) with CaCl_2_ and MgCl_2_ (Gibco, Life Technology Corp., Grand Island, NY) to remove unattached planktonic bacteria. Using sterile forceps, the mesh was then transferred to a new 35 mm polystyrene Petri plate and mounted using silicone sealer according to procedure outlined in Stoodley et al. 2012 [17]. One hundred *μ*L of LIVE/DEAD® Baclight™ Bacterial Viability stain was added to completely wet the mesh, according to the manufacturer's recommendations. The mesh was then placed in the dark for 25 minutes at room temperature. The stain was then removed and the mesh was washed once with 5 mL of HBSS. The plates were filled with 5 mL of HBSS. The biofilm on the mesh was imaged by confocal microscopy using a Leica DM RXE upright microscope attached to a TCS SP2 AOBS confocal system (Leica Microsystems, Exton, PA) using 5x or 63x water immersion objectives. Using ComStat Image Analysis software, living and dead microorganisms were counted and biofilms were quantified on the basis of biomass (um^3^/um^2^), average thickness (*μ*m), and substratum coverage (%).

### 2.5. Biofilm Analysis by Plate Count

After 24 hours of incubation, six plates containing bacterial inoculums samples (n=3) and control samples (n=3) were removed to perform plate count of recovered CFUs from formed biofilms and planktonic bacteria. To obtain planktonic bacteria CFUs, the broth culture was removed from the incubated mesh. Serial dilutions and drop pipetting onto BHI plates (five 10 *μ*L aliquots from a dilution series) were performed on the broth culture to obtain planktonic bacteria counts. To obtain bacterial biofilm CFUs, the mesh samples were rinsed three times with HBSS to remove unattached planktonic microorganisms. The mesh was then submerged in 2 mL of HBSS in a 15-mL polystyrene conical Falcon centrifuge tube. To detach and dissociate biofilm bacteria from the mesh, we used three cycles of a 10 s vortex followed by a 5 minute-sonication in a sonicator bath (Cole Palmer, Vernon Hill, IL). Bacteria were enumerated by drop pipetting five 10 *μ*L aliquots from a dilution series onto BHI plates. Experiments were performed in triplicate. After 24 hours of incubation at 37°C, the CFUs were counted for the appropriate dilution. Data was converted to CFU per cm^2^ (CFU/cm^∧^2^−1^) of mesh for formed biofilm and CFU per mL (CFU/mL^−1^) for planktonic bacteria. Data was reported as the geometric mean and 1 standard deviation calculated from on the basis of replicate drops and experiments, that is, n=6 for triplicate experiments.

## 3. Results

### 3.1. Biofilm Analysis by Confocal Microscopy

**Figure 1:**
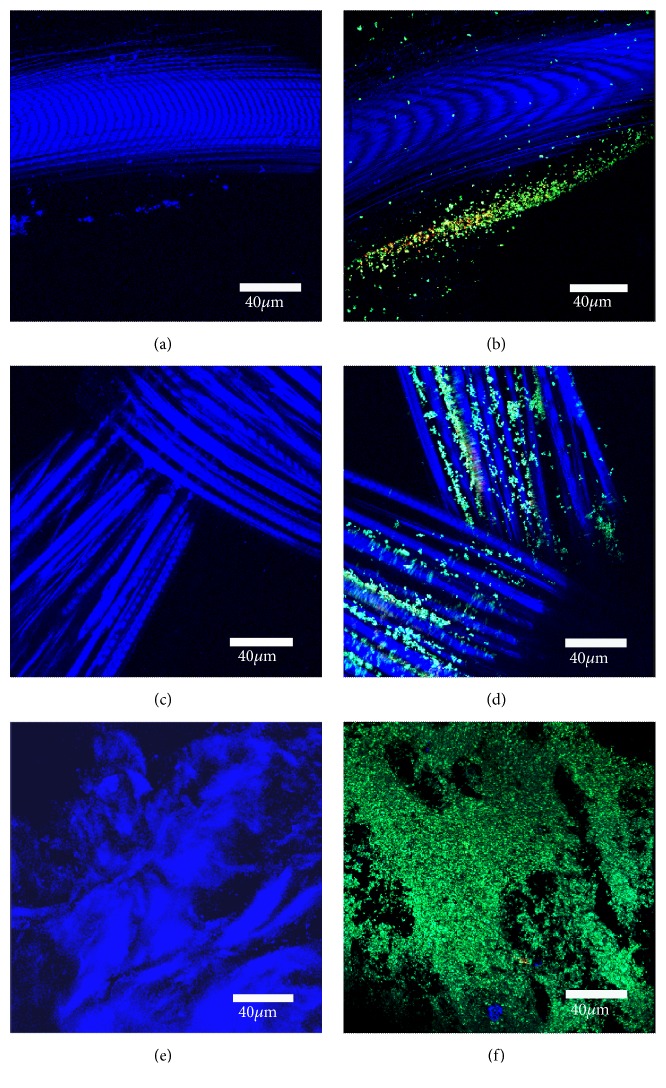
Polypropylene mesh (Images (a)-(b)), polyglactin 910 mesh (Images (c)-(d)), and biologic mesh (Images (e)-(f)) stained with LIVE/DEAD® Baclight™ Bacterial Viability stain and imaged using confocal microscopy. Images (a, c, and e) are mesh following 24 hr incubation with BHI while images (b, d, and f) are mesh after 24 hr incubation with* Staphylococcus aureus*. Live bacteria are stained green while blue represents reflective light from the mesh.

**Figure 2:**
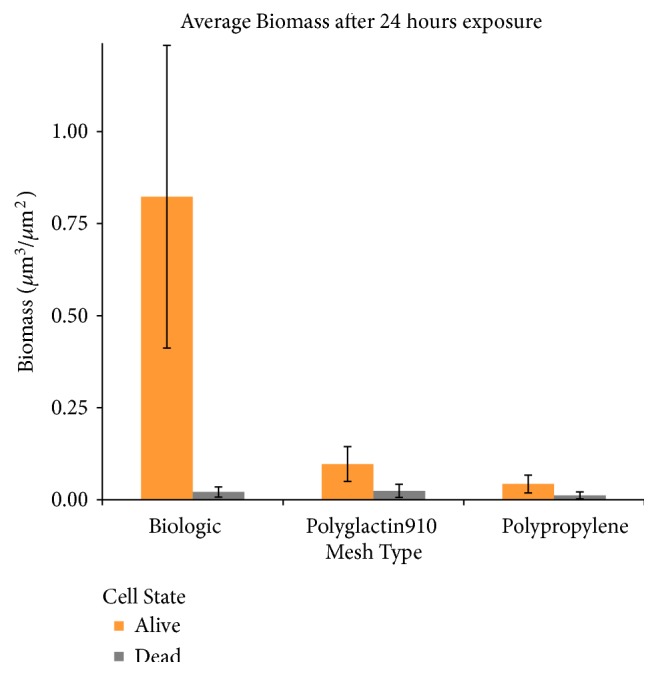
*Average Biomass after 24 hr Exposure to MRSA*. Quantification of biofilm biomass, orange bars represent viable bacteria and light grey bars nonviable bacteria.

**Figure 3:**
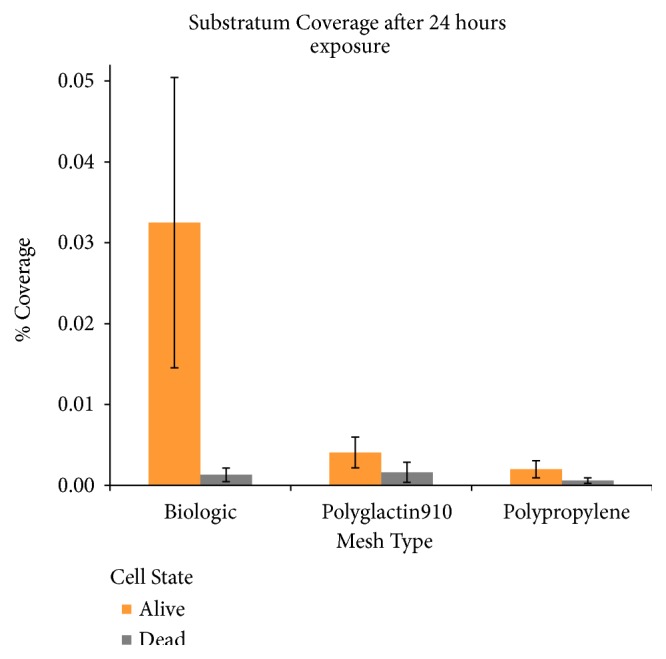
*Substratum Coverage after 24 hr Exposure to MRSA.* Quantification of biofilm substratum coverage, orange bars represent viable bacteria and light grey bars nonviable bacteria.

**Figure 4:**
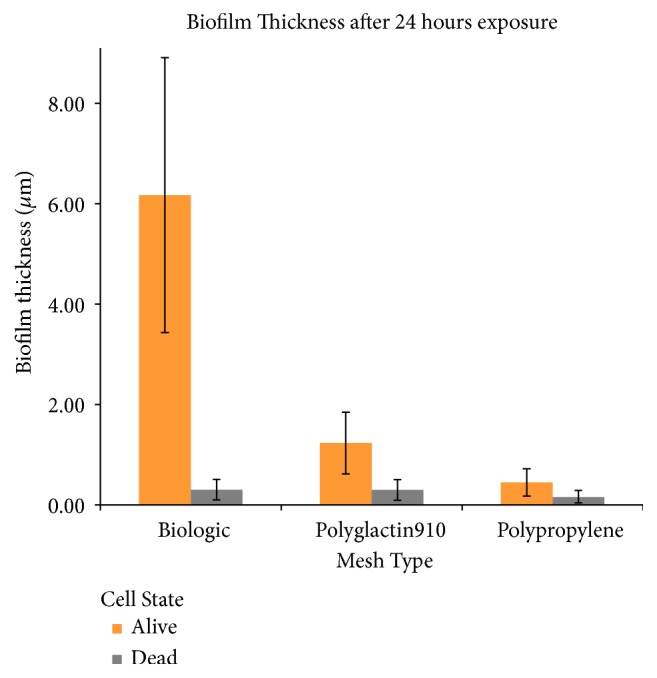
*Biofilm Thickness after 24 hr Exposure to MRSA. *Quantification of biofilm thickness, orange bars represent viable bacteria and light grey bars nonviable bacteria.

Each individual mesh was susceptible to bacterial growth (Figure 1) when visualized with LIVE/DEAD stain. However, based on visual inspection, it is evident that intact biofilms formed extensively on the human biologic mesh when compared to the synthetic mesh* in vitro*. Results of Comstat Image Analysis are shown in Figures 2–4. The biologic mesh had larger substratum coverage, thickness, and percent coverage when compared to both polypropylene and polyglactin 910 synthetic meshes.

### 3.2. Biofilm Analysis by Plate Count

We enumerated the attached bacteria as CFUs following mechanical detachment and dissociation of the biofilms from the meshes, completed using successive vortex/sonication cycles. Plate counts of formed biofilm were significantly greater for the human biologic compared to the synthetic meshes.

**Figure 5:**
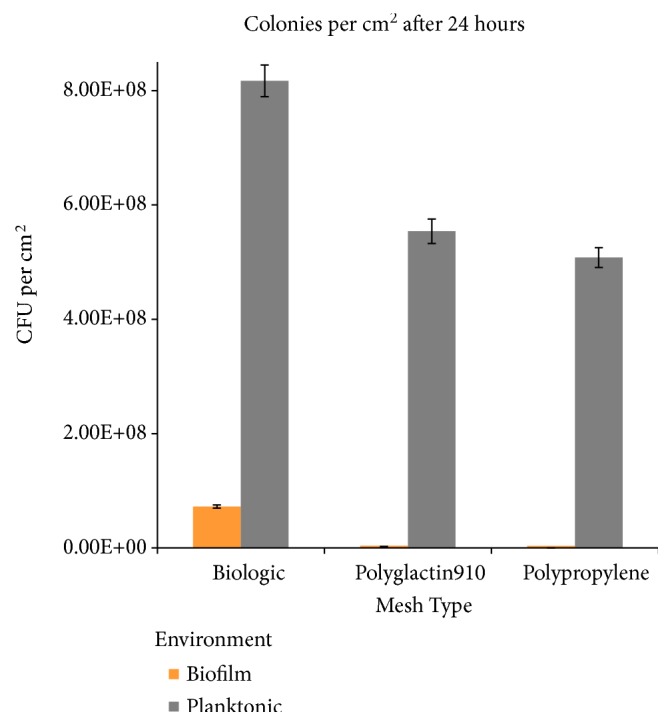
*Quantification of Colony-forming Units per cm* ^*2*^ *after 24 Hours.* Dark grey bars represent attached biofilm bacteria and light grey bars planktonic bacteria.

All analyses were carried out in triplicate, and the results are expressed as the average of the assays. The attached bacteria per cm^2^ of mesh averaged 7.25 × 10^7^ for human biologic compared to 1.65 × 10^5^ for PPM and 1.04 × 10^6^ for polyglactin 910. Results of plate counts for formed biofilm and plate counts for planktonic bacteria for the different mesh types are shown in Figure 5. The amount of adhered bacteria is significantly greater on the biologic mesh vs. both polyglactin 910 and polypropylene mesh.

## 4. Discussion

While synthetic meshes have undoubtedly been effective in reducing inguinal hernia recurrence rates [3–5, 21], there are complications that correlated with their use including adhesion, fistulas, and most notably infection [7]. With mesh-related infection rates of 9% reported for open inguinal hernia repair [22] and open incisional hernia repair infection rates as high as 18% [10], complications from mesh repair remain significant. In fact, further development of biologic meshes for abdominal wall reconstruction was mainly inspired by the persistence of complications, including infection, following synthetic mesh implantation [2]. As such, the sharp rise in popularity of biological meshes has been largely catalyzed by the common belief that “biological meshes […] are more resistant to infection” [18].

Indeed, due to concerns of infection with synthetic mesh implantation, many surgeons have turned to biologic meshes to repair complex abdominal wall defects, especially in contaminated fields [23]. Speculation that biologic mesh “yields low infection rates” [2] largely stems from the capacity of biologic meshes to, upon implantation, become quickly and easily integrated into host tissue as a result of rapid neovascularization, collagen deposition, site-specific tissue remodeling, and tissue ingrowth [18, 24]; the theory behind these findings is that following scaffold incorporation by the host, the host tissue and host immune system are brought close to the biomaterial surface, thereby enhancing protection of the mesh from microorganisms [25].

However, despite the growing popularity, prevalent use, and significant associated costs of biologic mesh [26], there is minimal data backing the widespread notion that biological grafts are more infection resistant. Current data on this matter remains mixed and inconclusive. In a large multistudy review comparing biologic mesh to synthetic mesh for ventral hernia repair, researchers found that biologic meshes had a significantly lower incidence of infectious wound complications compared to synthetic meshes [27]. Conversely, a review by Sandvall et al. comparing biologic and synthetic meshes for use in ventral hernia repair discovered the opposite. Patients in the biologic group had significantly greater major complication rates (22% vs. 15%), significantly greater minor complication rates (37% vs. 26%), and significantly greater recurrence rates (11% vs. 5%) when compared to patients in the synthetic group [28]. Moreover, Harth and Rosen have shown that use of biologic prosthetics to repair complex abdominal wall defects has resulted in a myriad of infectious complications [29].

With 20 million meshes being implanted worldwide each year [1], the lack of conclusive data about the anti-infective properties of biologic mesh combined with the high rate of postsurgical mesh infection is particularly concerning. As such, it is as important to understand the interplay between prosthetic biomaterials and infectious complications. While clinical research comparing biologic and synthetic meshes based on clinical complications is important, we believed it is equally important to investigate and compare meshes in an infectious* in vitro* model to better understand this issue.

Upon examination of the results, this experiment illustrates that biofilms formed on biologic mesh* in vitro *were significantly larger and thicker and covered more of the substratum when compared to the synthetic meshes. Several studies have shown that initial bacterial adhesion and biofilm formation are dependent on material characteristics such as hydrophobicity, porosity, and filament number [16, 17, 30]. Therefore, we believe that our results can largely be attributed to the inherent morphological aspects of biologic mesh. Studies of surface morphology of biologic prosthetics by Bellows et al. [26] found that the rough surfaces and niches in biologic meshes provide more contact points that enable adhesion of bacteria and similarly they found that bacteria are more easily trapped in between the collagen fibers of the biologic mesh [23]. Our results support these findings that the surface and material properties of biological meshes promote bacterial attachment and biofilm formation* in vitro*.

Ultimately, the results from this study illustrate both synthetic meshes ability to resist single-species MRSA biofilm formation* in vitro* more effectively than biologic mesh. However, replication of these experiments in a relevant* in vivo* model is needed to fully understand the relationship between surgical mesh type and infection. Perhaps, an* in vivo* model will control mesh “behavior” after implantation, such as the perceived characteristic of biologic mesh to mimic host tissue theoretically making it less prone to infection. In addition, an* in vivo* model is needed to control for various other related factors, such as host immune response, immunosuppression, and the implantation environment that all contribute to the pathogenesis of mesh-related infection.

## 5. Conclusion

To our knowledge, this study is the first that comparatively and directly examines single-species biofilm formation on biologic and synthetic surgical meshes. We believe this work to be important and clinically significant, as it should help reduce some of the obscurity surrounding the major problem of prosthetic mesh infection. Given the ever-increasing number of commercially available surgical meshes and the fact that we tested a relatively small sample size, further research should include various other commercially available synthetic and biologic meshes, tested in both* in vitro* and* in vivo* models. Ultimately, obtaining a more complete understanding of the relationship between biomaterials and infectious complications has significant implications for the selection of biomaterials by surgeons in clinical settings, as well as for future scientists who seek to minimize mesh-related complications by developing a more superior prosthetic biomaterial.

## Data Availability

The data used to support the findings of this study are available from the corresponding author upon request.
